# DIAGNOSTIC CHALLENGES IN DISTINGUISHING AUTISM SPECTRUM DISORDER FROM PSYCHOSIS: A CASE REPORT

**DOI:** 10.1192/j.eurpsy.2023.2321

**Published:** 2023-07-19

**Authors:** W. W. S. J. Lee, S. Y. L. Lee

**Affiliations:** Institute of Mental Health, Singapore

## Abstract

**Introduction:**

Autism spectrum disorders (ASD) and psychotic disorders have historically considered to be related conditions with a long history of diagnostic confusion. Although DSM-III distinguishes ASD and Schizophrenia Spectrum Disorders as distinct clinical entities, they continue to share overlaps in their clinical symptom presentations leading to diagnostic challenges that may consequentially result in delayed treatment. Prompt diagnosis is crucial in the context of psychosis, where early intervention impacts recovery.

**Objectives:**

To present the diagnostic challenges encountered in distinguishing ASD from Psychosis.

**Methods:**

We present a case report demonstrating the challenges of distinguishing ASD from Psychosis.

**Results:**

This is a case of a gentleman who initially presented to psychiatric services at 18 years old for conflicts with his mother related to his inflexibility to change. Further psychological evaluation revealed that he had a history of restricted social interaction with his peers, difficulties in non-verbal communications and identifying emotional states, stereotyped interests and obsessions that isolated him from his peers. He was diagnosed with ASD.

In subsequent presentations, there were symptoms of excessive preoccupation of his facial appearance, excessive concern over contracting HIV, obsessions with arranging objects in a particular order and avoiding words starting with the letter “S” out of fears of blasphemy. While these symptoms had qualities of cognitive inflexibility, they could not fully be explained by ASD. Additional diagnoses of Body Dysmorphic Disorder, Borderline Personality Disorder, Obsessive Compulsive Personality Disorder and At-Risk Mental State were considered.

A psychiatric admission was necessitated at 21 years old, when he presented with a 2-year history of repetitive banging of furniture in the middle of the night to communicate his frustrations towards his parents for their perceived acts of blasphemy. He also began to isolate himself, fearing that his parents would be able to look into his soul and reveal his sins. This paranoia towards his parents worsened to the point of urinating and defecating in his room to avoid his parents. His school performance declined as well.

A unifying diagnosis of psychosis was made. His previous diagnosis of ASD was challenged as a misdiagnosis, with the impression that he likely had attenuated psychotic symptoms in his adolescent years, disguised as autistic traits. The diagnosis of psychosis was confirmed when the patient’s symptoms were observed to respond to antipsychotic treatment.

**Conclusions:**

This case report illustrates the challenges in distinguishing ASD from psychosis. A prior diagnosis of ASD may result in diagnostic overshadowing and subsequent delays in diagnosing psychosis. Further research in diagnostic tools would be helpful for diagnostic precision, thereby enabling prompt treatment for better recovery outcomes.

**Disclosure of Interest:**

None Declared

